# Normative reference values for estimated cardiorespiratory fitness in apparently healthy British men and women

**DOI:** 10.1371/journal.pone.0240099

**Published:** 2020-10-08

**Authors:** Lee Ingle, Alan Rigby, David Brodie, Gavin Sandercock

**Affiliations:** 1 Department of Sport, Health & Exercise Sciences, University of Hull, Hull, United Kingdom; 2 Hull York Medical School, University of Hull, Hull, United Kingdom; 3 Department of Health, Buckinghamshire New University, Wycombe, Bucks, United Kingdom; 4 Centre for Sport & Exercise Science, University of Essex, Essex, United Kingdom; Univ Rennes, FRANCE

## Abstract

**Objectives:**

To develop normative reference standards for estimated cardiorespiratory fitness (eCRF) measured from treadmill-based incremental exercise testing in ~12 000 British men and women.

**Methods:**

Cross-sectional study using retrospectively collected eCRF data from five preventative health screening clinics in the United Kingdom. Reference centiles were developed using a parametric approach by fitting fractional polynomials. We selected the ‘best’ powers by considering both the smallest deviance, and clinical knowledge from the following set of *a priori* decided powers (-2,-1,-0.5, 0, 0.5,1,2,3). A series of fractional polynomials (FPs) were investigated with three-parameters (median, standard deviation and skewness). The following reference centiles were plotted (3, 5, 10, 25, 50, 75, 90, 95, 97).

**Results:**

We included 9 204 males (median [25^th^,75^th^ centiles] age 48 [44, 53] years; BMI 27 {25, 29] kg∙m^-2^; peak VO_2_ 36.9 [30.5, 44.7] ml∙kg^-1^∙min^-1^) and 2 687 females (age 48, [41, 51] years; BMI 24 {22, 27] kg∙m^-2^; peak VO_2_ 36.5 [30.1, 44.8] ml∙kg^-1^∙min^-1^) in our analysis to develop the normative values.

**Conclusion:**

Reference values and nomograms for eCRF were derived from a relatively large cohort of preventative health care screening examinations of apparently healthy British men and women. Age- and sex-specific eCRF percentiles were similar to data from international cohort studies. The adoption of submaximal exercise testing protocols reduces individual risk when exercise history is unknown and testing is conducted in a community-based setting. Our findings can be used by health professionals to help guide clinical decision making.

## Introduction

Robust epidemiological evidence has consistently shown a strong association between low cardiorespiratory fitness (CRF), and a higher incidence of disease risk including some cancers, cardiovascular disease, and metabolic syndrome, amongst other conditions [1–3]. Adults with low CRF have a greatly increased risk of premature all-cause and cardiovascular mortality compared to individuals with the highest levels of CRF [4]. Objectively measured CRF ‘outperforms’ traditional risk factors as an indicator of health [5], and as a predictor of all-cause and cardiovascular mortality [6]. Despite its high predictive power, CRF has not been included in widely used cardiovascular risk models such as Framingham [7], or JBS3 [8]. These studies provide strong evidence to support public health messages emphasising the importance of improving CRF for promoting cardiometabolic health. Unlike US guidelines [9], current UK messages largely focus on the promotion of habitual physical activity *per se*, and in many cases, the relatively weak association it shares with weight status and weight gain.

In 2003, both the American Thoracic Society and the American College of Chest Physicians emphasised that valid and representative reference values were critical for the interpretation of CRF [10]. Only in recent years have registries from the US, Norway, Germany, and Brazil published normative reference values for CRF [11–17]. However, in the UK, despite the potential value of exercise testing in health surveillance and preventive healthcare, objectively measured CRF is not routinely assessed in health surveys. Objective measures of CRF may provide important diagnostic and prognostic value, particularly when derived from the “gold standard” modality, maximal cardiopulmonary exercise testing [18]. Objective measures of CRF may be only moderately correlated with estimated CRF (eCRF) [19], however, in the UK, objectively measured CRF testing is rarely undertaken in health surveillance and preventive healthcare, therefore investigating alternative methods for estimating CRF would seem prudent. In recent years, there has been a call to consider eCRF as a patient ‘vital sign’, and thus, incorporate its inclusion into routine patient health assessment [20]. The value of eCRF as an important risk prognosticator has been recently reported in higher risk populations [21].

Few data reporting objective or estimated CRF data in a British population exist [22], and, indeed, we are not aware of any published national reference standards for CRF in British men and women. The aim of the study was to generate age- and sex-specific nomograms for estimated CRF values in a sample of ~ 12,000 men and women from preventive health care consultations across five non-medical sites in the United Kingdom. A secondary aim was to compare and contrast our findings with established CRF registries which have used maximal or symptom-limited testing protocols.

## Methods

Ethical approval was granted by the Faculty of Society & Health ethics committee, Buckinghamshire New University. Males and females (aged 19–80 years) attended one of five Health & Wellbeing clinics around Great Britain for a three-hour preventative health assessment. Participants attended general health examinations as an annual benefit provided by their corporate wellness schemes. Screening attendance was voluntary, as such the study participants represent a self-selected opportunity sample. Each participant was instructed in their pre-assessment information pack to avoid vigorous physical activity, alcohol and caffeinated beverages for 24 hours prior to their assessment. Participants, in a supine position, underwent a resting electrocardiogram (ECG) for 5 min using the Marquette CASE Stress system (GE Healthcare, UK). Each participant signed and consented to the test battery which was countersigned by the duty medical officer.

### Demographic and anthropometric measurements

Body mass was measured using digital scales (Marsden Weighing, Rotherham, UK) and recorded to the nearest 0.1 kg. Clothing was worn but shoes and belts were removed, and participants evacuated their bladder before stepping onto the scales. Scales were calibrated daily with a known weight and bi-annually by the manufacturer. Stature was measured using a stadiometer (Seca, Hamburg, Germany) and recorded to the nearest 0.1 cm. Participants removed their shoes, stood on the platform with feet together, and head in the Frankfort plane. Buttocks and scapulae were in contact with the back of the stadiometer, shoulders relaxed with hands and arms loosely at the sides, the measurement was taken on full inhalation. Waist circumference (WC) was measured to the nearest 0.1 cm using a flexible anthropometric tape measure, midway between the lowest rib and the iliac crest at minimal inspiration.

### Venous blood sampling

Participants presented in a fasted state (for the previous 12 hours) but ate a snack (fruit or muesli bar) prior to the exercise test. At the start of each assessment, fasted venous blood samples was obtained using vacutainer tubes and heparinised whole blood was analysed using the Piccolo blood chemistry analyser (Abaxis, USA). The following analytes were measured: glucose, total cholesterol (TC), low density lipoprotein (LDL), high density lipoprotein (HDL), triglycerides, and TC/HDL ratio.

### Estimated CRF

Resting blood pressure was measured using a manual system (Accoson Duplex Aneroid Model, AC Cosser & Son Ltd, UK). Participants positioned themselves on a T2100 treadmill (GE Healthcare, UK), and undertook an incremental exercise test using the Bruce protocol [23]. Blood pressure was monitored at the second minute of each stage using the automatic Tango stress test BP monitor (Suntech Medical, Oxfordshire, UK). The ECG was monitored throughout the test. Participants exercised until they attained ~85% of age-predicted maximum heart rate (220-age) or met any of the test termination criteria outlined by the American College of Sports Medicine [24]. VO_2peak_ was estimated and reported relative to body mass (ml·kg^-1^·min^-1^) [23].

## Statistical analysis

### Historical perspective

Reference centiles are routinely used in clinical decision making. Statistical methods for developing them include the LMS method (L = skewness, M = median, S = coefficient of variation) which is widely used for measuring growth in children [25, 26]. LMS is a semi-parametric technique, but as with all semi-parametric approaches it is difficult to articulate explicit formulae for centile estimates at a given age. Age groupings are subjective, and centile curves can vary accordingly. Others have developed parametric approaches [27]. The Wright and Royston approach [27] fits fractional polynomials (FPs) to the parameters associated with the reference centiles. FPs give a wider range of shapes than conventional polynomials. These two techniques for centile estimation are the main methodologies available [28]. A range of centile curves may describe the data equally well with final selection down to personal choice (smoothness of curve, simplicity, clinical knowledge), and not necessarily the one with the smallest deviation (calculated as minus twice the log-likelihood).

### Peak oxygen uptake transformation

The normal distribution was adopted as our starting point for centile estimation owing to its mathematical simplicity, understanding and general usefulness. Shapiro-Wilk was used to test for departures from normality. In males, VO_2_peak did not follow a normal distribution (*P*<0.001) using Shapiro-Wilk. When data has a positive skew a log-transformation will reduce skewness. Under a log-10 transformation skewness was significantly reduced (*P* = 0.29). A plot of the scaled absolute residuals vs age was almost a straight line; this indicated that there was evidence of increasing standard deviation with age. In females, VO_2_peak was skewed (*P*<0.001) using a Shapiro-Wilk test. Skewness was not statistically significant under a log-10 transformation (*P* = 0.70).

### Fractional polynomials (FP)

FP regression was used to develop reference centiles for men (n = 9 204) and women (n = 2 687) separately. A series of FPs were investigated with three-parameters (median, standard deviation and skewness). FP regression produces a table of regression model comparisons by searching through the *a priori* power combinations outlined above. Goodness-of-fit of the FP regression models was carried out by plotting histograms of the Z-scores. Further verification was by q-norm plots of standard deviations (SDs) [29]. Q-norm plots were then compared to the distribution of the SDs, with a straight line indicating adequacy. The following reference centiles were plotted (3, 5, 10, 25, 50, 75, 90, 95, 97).

A statistical comparison between males and females was made by independent t-test (Table 1). A Pearson’s Chi-squared test was used to compare binary data. An arbitrary level of 5% statistical significance (2-tailed) was assumed.

**Table 1:** Demographical data separated by gender (mean; 95% confidence intervals).

	Males	Females	*P*-value
Body fat (%)	23(20,26)	28(24,33)	<0.001
Waist circumference (cm)	103(99,108)	99(95,105)	<0.001
SBP (mmHg)	124(118,133)	118(110,126)	<0.001
Glucose (mmol∙l^-1^)	5(5,6)	5(5,6)	0.65
Total cholesterol (mmol∙l^-1^)	3.7(3,4.5)	3.6(2.9,4.5)	0.28
LDL cholesterol (mmol∙l^-1^)	3(3,4)	3(3,4)	0.37
HDL cholesterol (mmol∙l^-1^)	1(1,2)	1(1,2)	0.11
Triglycerides (mmol∙l^-1^)	1.1(0.8,1.6)	1.1(0.8,1.6)	0.85

SBP: systolic blood pressure

## Results

We included 9 204 males (median [25^th^, 75^th^ centiles] age 48 [44, 53] years; BMI 27 {25, 29] kg∙m^-2^; peak VO_2_ 36.9 [30.5, 44.7] ml∙kg^-1^∙min^-1^) and 2 687 females (age 48 [41, 51] years; BMI 24 {22, 27] kg∙m^-2^; peak VO_2_ 36.5 [30.1, 44.8] ml∙kg^-1^∙min^-1^) in our analysis (Table 1). Males had higher mean BMI, waist circumference, resting systolic blood pressure (all *P*<0.001), and mean peak oxygen uptake compared to females (*P*<0.05). Females had higher mean % body fat compared to males (*P*<0.001). All measured blood biomarkers were not different between males and females (*P*>0.05).

### Model comparison

There were no significant differences between any of the FP regressions for either males (Table 2) or females (Table 3), indicated by the ‘difference from the lowest deviance’ statistic. The ‘best fit’ model statistically is the one with the lowest deviance. We selected mean powers (-0.5, 0 males) and (-0.5,-0.5) females; SD powers (1) for both. Model diagnostics were adequate for both sexes. Z-scores followed an approximate normal distribution. Q-norm plots of SDs were on a straight line. Tables 2 and 3 showed a number of plausible models. We compared powers (-0.5, 0 males) and (-0.5, -0.5 females) and their linear models (3^rd^, 50^th^, 97^th^ centiles for illustration)). In males, a straight line closely matched those developed by FP. In females, the largest discrepancy *within centiles* was seen in the highest centile. This was observed in both younger and older women. In the lower centiles, less marked but similar patterns were found. Model diagnostics were adequate for both sexes.

**Table 2:** Fractional polynomial regression in males.

Age	DF	Model deviance statistic	Deviance difference	P-value	Powers
Omitted	0	-13588.98	1.77	0.77	
Linear	1	-13590.38	0.48	0.92	1
M = 1	2	-13590.70	0.06	0.97	3
M = 2	4	-13590.76	0.00	Reference	-0.5, 0

The linear model (second row) is default. The degree-of-freedom column headed ‘DF’ shows the number of additional parameters used in each FP model beyond the quantity of parameters in the null model. The deviance statistic (-2 log likelihood) is given in the column headed ‘Model deviance statistic’. The difference in deviances from the lowest value is given in the column headed ‘Deviance difference’. The *P*-value comparison of these differences is shown in the penultimate column. Powers are given in the last column headed ‘Powers’. Statistically, the model with the ‘best fit’ is the one with the lowest deviance; here this model has powers (-0.5, 0). Note that none of the FPs were statistically significant from the model with the lowest deviance.

**Table 3:** Fractional polynomial regression in females.

Age	DF	Model deviance statistic	Deviance difference	P-value	Powers
Omitted	0	-3838.82	1.68	0.79	
Linear	1	-3838.87	1.62	0.65	1
M = 1	2	-3838.01	1.48	0.47	3
M = 2	4	-3840.50	0.00	Reference	-0.5,-0.5

The selection of powers in females followed the same process as that for males. The model with the lowest deviance has powers (-0.5,-0.5). There were no significant differences between the lowest deviance model and the other FP regression models. Model diagnostics were satisfactory. Z-scores followed an approximate normal distribution. A q-norm plot of the SDs was on a straight line. Centile curves are plotted in Fig 2. Top centile curves (90^th^-97^th^) peaked in the 30–40 year old age groups but were flatter at the bottom end (3^rd^ centile). After 30–40 years of age centile curves declined.

**Fig 1:**
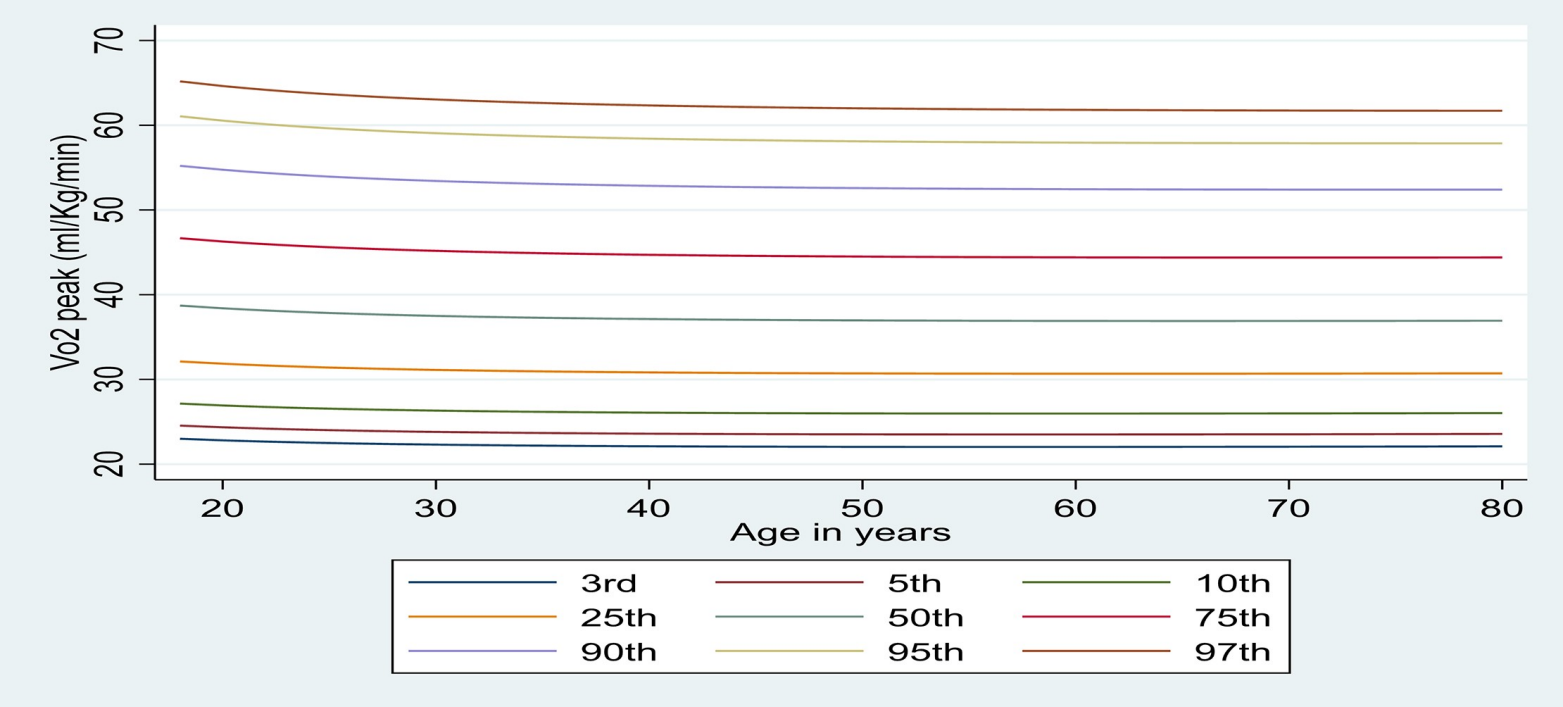
Peak VO_2_ (ml∙kg^-1^∙min^-1^) centile reference curves in apparently healthy British males.

**Fig 2:**
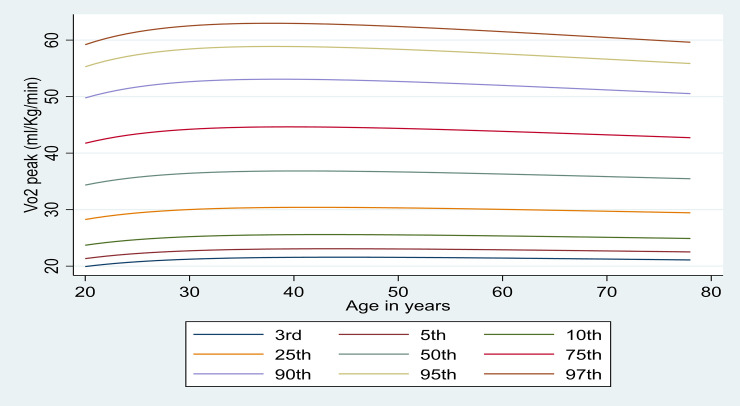
Peak VO_2_ (ml∙kg^-1^∙min^-1^) centile reference curves in apparently healthy British females.

Figs 1 and 2 illustrate centile curves for males and females, respectively. In males, curves showed a steady decline until 65–70 years, no matter what the centile. The pattern was different in females where women aged 30–40 years peaked in the upper centiles especially, and fell away quickly.

### Data comparison with other international CRF registries

**Fig 3:**
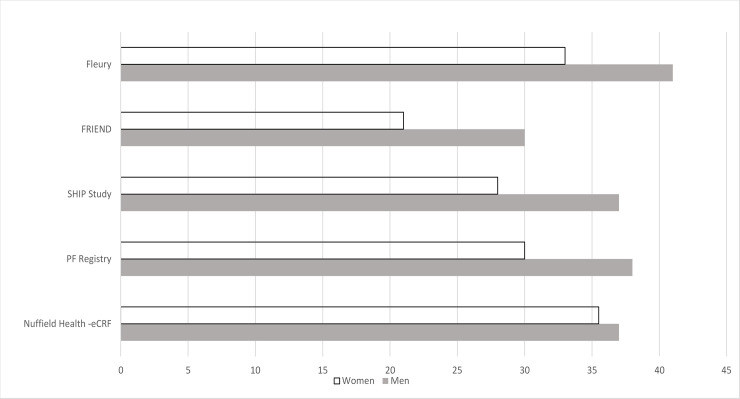
Comparative values of normative aerobic fitness data (peak VO_2_ in ml∙kg^-1^∙min^-1^) from Nuffield Health (estimated CRF) and other registry findings including Fleury, FRIEND, SHIP, and PF Registry for a 45-year-old male and female in the 50^th^ percentile range.

A secondary aim of the study was to compare our estimated CRF data with data derived from other registries using maximal or symptom-limited testing protocols (Table 4). Fig 3 shows comparative data from different international registries for peak oxygen uptake in apparently healthy men and women. Exercise test modality was either treadmill or cycle ergometry. For a direct comparison, we used a 45-year old male and female and compared 25^th^, 50^th^ and 75^th^ percentile ranges. In the 25^th^ percentile range: CRF ranged from 23–34 ml∙kg^-1^∙min^-1^ in men across the five registries with the Nuffield Health data being the second highest (31 ml∙kg^-1^∙min^-1^) behind the Fleury registry which used a maximal intensity treadmill protocol.^16^ In women, CRF ranged between 20–30 ml∙kg^-1^∙min^-1^ dependent upon the registry with the Nuffield Health data producing the highest value (30 ml∙kg^-1^∙min^-1^). In the 50^th^ percentile category: CRF ranged from 36–41 ml∙kg^-1^∙min^-1^in men (Nuffield Health 37 ml∙kg^-1^∙min^-1^), and in women ranged between 26–36 ml∙kg^-1^∙min^-1^, with Nuffield health data being the highest (36 ml∙kg^-1^∙min^-1^; Fig 3). In the 75% percentile category: CRF ranged between 43–48 ml∙kg^-1^∙min^-1^ in men (Nuffield Health data was towards the lower end: 44 ml∙kg^-1^∙min^-1^) and, in women, between 38–44 ml∙kg^-1^∙min^-1^ (Nuffield-Health was the highest value: 44 ml∙kg^-1^∙min^-1^). Our findings indicate strong similarities between the estimated CRF values from Nuffield Health data and international registries which have used maximal or symptom-limited testing protocols.

**Table 4:** Comparison between selected international fitness registry data and eCRF from Nuffield Health.

Country of Origin	Sample (n =?)	Registry	Exercise Mode	Exercise Intensity	25^th^ Percentile (ml∙kg^-1^∙min^-1^)*	50^th^ Percentile (ml∙kg^-1^∙min^-1^)*	75% Percentile (ml∙kg^-1^∙min^-1^)*
Germany	10,090	PF Registry	Cycle ergometry	Maximal. Peak RER>1.10	Men: 25	Men: 38	Men: 48
					Women: 20	Women: 30	Women: 41
Germany	1,708	SHIP	Cycle ergometry	Symptom-limited. Maximal effort encouraged	Men: 23	Men: 37	Men: 47
					Women: 22	Women: 28	Women: 40
United States of America	7,783	FRIEND	Treadmill	Maximal encouraged. Peak RER>1.00	Men: 30	Men: 36	Men: 43
					Women: 21	Women: 26	Women: 31
Brazil	18,189	Fleury	Treadmill	Maximal. Peak RER>1.10	Men: 34	Men: 41	Men: 45
					Women: 28	Women: 33	Women: 38
United Kingdom	11,891	Nuffield Health	Treadmill	Submaximal (85% age-predicted maximum predicted HR)	Men: 31	Men: 37	Men: 44
					Women: 30	Women: 36	Women: 44

*Estimated values derived from available data. Percentile ranges based on a 45-year-old male/female in the 25^th^, 50^th^ and 75% percentile range for directly determined and estimated cardiorespiratory fitness using either a treadmill or cycle ergometer test modality.

The eCRF findings in females were generally at the higher end of the range compared to data from international registries, which may not be too surprising as both SHIP and the PF Registry used cycle ergometry as the mode of exercise, and our study used a treadmill protocol [15, 17]. In men, estimated CRF from Nuffield Health data varied between the upper and lower end of the range compared to international registries which used maximal intensity protocols. Overall, in our example, there was less variability between international registries and Nuffield Health data in higher fitness categories compared to lower ones. For Nuffield Health data, female data was generally towards the higher end compared to international registries, therefore, it was generally closer to comparative male data (using identical percentile ranges) compared to international registries.

## Discussion

Our study is the first to report age- and sex specific normative reference values for estimated CRF in British men and women. The sample includes ~12,000 participants who underwent preventative health screening in five non-medical community centres in the UK. The modality of exercise testing will strongly influence the peak oxygen uptake values obtained and therefore the normative reference values produced. Peak oxygen uptake measured by treadmill ergometers may be >10% higher compared to testing conducted on cycle ergometry due to the recruitment of larger muscle groups and test termination is less likely to be due to the effects of local muscular fatigue [10]. In both the PF Registry (n = 10.090), and SHIP (n = 1,708) fitness registries which emanate from Germany, testing was conducted on a cycle ergometer [15, 17]. In contrast, the FRIEND registry (n = 7,783) which originates in the USA [12], used treadmill-based exercise testing, as did the Fleury registry (n = 18,189) from Brazil 16].

The other major factor which is likely to account for differences in fitness outcomes is whether the testing protocol elicited maximal responses from the participants. The testing protocols of the PF Registry and the Fleury study elicited maximal responses using an objective physiological threshold of peak respiratory exchange ratio >1.10 [15, 16]. However, other registries including SHIP used a symptom-limited protocol where maximal effort was encouraged, and FRIEND encouraged maximal effort, although they set a lower threshold for peak respiratory exchange ratio of >1.00 [12, 17]. Therefore, it is not certain whether participants in the FRIEND and SHIP registries achieved the criteria for maximal intensity exercise [12, 17]. The Nuffield Health registry data incorporated estimated CRF data (based on 85% age-predicted HR maximum), however, eCRF has been shown to be only moderately correlated with objectively measured CRF [19]. Nuffield Health data was collected between 2000–2009. Between 2000–2006, participants were encouraged to perform a maximal exercise test to volitional exhaustion. However, between 2006–2009, the protocol changed, and test termination criteria of achieving 85% predicted maximum heart rate was introduced. There was no statistical difference between the measured and predicted values, indicating that the change in protocol was not associated with systematically different estimates of CRF.

It is interesting to speculate on the shape of the CRF curves produced; we found a significant difference in mean peak oxygen uptake between males and females. It has been well established that females have relatively smaller lungs and conducting airways than males [30]. During incremental exercise, females develop greater expiratory flow limitation and a greater work of breathing for a given minute ventilation compared to males [31], which may partly explain their relatively lower CRF values. We know that endurance training improves CRF, however, we are not aware of the training history of our participants. The male curves generally peak in the 20’s decade, and gradually declined or flattened in the older age strata. In females, the curves generally peaked around 35–40 years before falling away in the older strata. It is noteworthy, that of our entire sample size, only ~22% of the population was female, and this may be partly responsible for the different trends in CRF profiles.

The Nuffield Health data were similar to published findings from established registries using maximal intensity testing protocols showing the value of eCRF for age- and sex-adjusted normative fitness estimates in the British population. Whilst maximal exercise testing protocols are encouraged to optimise the validity of the data, we may be increasing patient risk and safety concerns by asking individuals to perform maximally without knowledge of their exercise history. In the UK, fitness testing is not routinely undertaken in the general population, and in those sectors where it is, submaximal testing is frequently used in order to minimise risk. Therefore, our findings using eCRF are more representative of the testing protocols likely to be conducted in the UK, and thus better reflect CRF profiles collected in health screening/health surveillance settings.

### Limitations

The study was not designed with centile estimation in mind, and sample size was not determined *a priori*. We acknowledge the relatively low sample size, especially in females for our reference centile estimation. Developing satisfactory statistical methods for estimating sample size for reference centiles is surprisingly difficult [32], and this has not changed much in recent years [33]. A common rule-of-thumb has been to use 50, 100, 200 per group. This assumes the same width for each age band which is unrealistic in real-life surveys. For normally distributed data, the median will have the best precision; conversely extreme centiles will be less precise. This is illustrated in our data. The median age (48 years) consisted of 1 839 participants, while at the lower extremity (≤20 years), consisted of seven participants. Precision was high (<1%) at the median but not at the lower end.

In conclusion, we have generated age- and sex-specific reference nomograms from estimated CRF values in a sample of more than 12,000 men and women from preventive health care consultations across five non-medical sites in the United Kingdom. Age- and sex specific eCRF percentiles were similar to data from international cohort studies using maximal or symptom-limited testing protocols. The adoption of submaximal exercise testing protocols reduces individual risk when exercise history is unkmown and testing is conducted in a non-medical community-based setting.

## Supporting information

S1 Data Data collected for this investigation. (CSV)
